# Supplementary material for: An allosteric switch between the activation loop and a c-terminal palindromic phospho-motif controls c-Src function
Source: Nat Commun. 2023 Oct 17;14:6548. doi: 10.1038/s41467-023-41890-7 (PMC10582172; doi:10.1038/s41467-023-41890-7)
Supplement: Supplementary file 1 — Supplementary information [file 41467_2023_41890_MOESM1_ESM.pdf]

Supplemental Information

An allosteric switch between the activation loop and a c-terminal palindromic phospho-motif controls c-Src function

Cuesta and Contreras et al.

1. Supplementary figures

Supplementary figure 1

(a-b) Drosophila Src42A purification and MS identification of oligomeric species

(c-d) Evaluation of Myr-G2 versus G2 peptides on binding and activity assays

Supplementary figure 2. SAXs analysis of an un-phosphorylated c-Src 3D-construct

Supplementary figure 3. MS data of c-Src WT auto-phosphorylation

Supplementary figure 4. MS data of c-Src WT, Y419F and Y530F auto-phosphorylation

Supplementary figure 5. Enzymatic activity (K_M ATP) at increasing enzyme concentration of c-Src WT, versus Y419F and Y530F mutants

Supplementary figure 6. MD simulation analyses of the assymetric c-Src dimer/c-terminal Tyr 530 auto-phosphorylation

Supplementary figure 7. Functional evaluation of c-Src D521A and 529X mutants

Supplementary figure 8. Enzyme kinetics of c-Src WT and the 531X using c-terminal derived peptides

Supplementary figure 9. Functional evaluation of a c-Src PAYAP palindrome mutant

Supplementary figure 10. Functional evaluation of phosphorylated c-Src and WBs analyses of tumor cell lines using total and phospho-specific c-Src antibodies

2. Supplementary tables

Supplementary Table 1. SAXs data collection and structural and mass parameter

3. Supplementary Methods

4. Supplementary references

1. SUPPLEMENTARY FIGURES

a

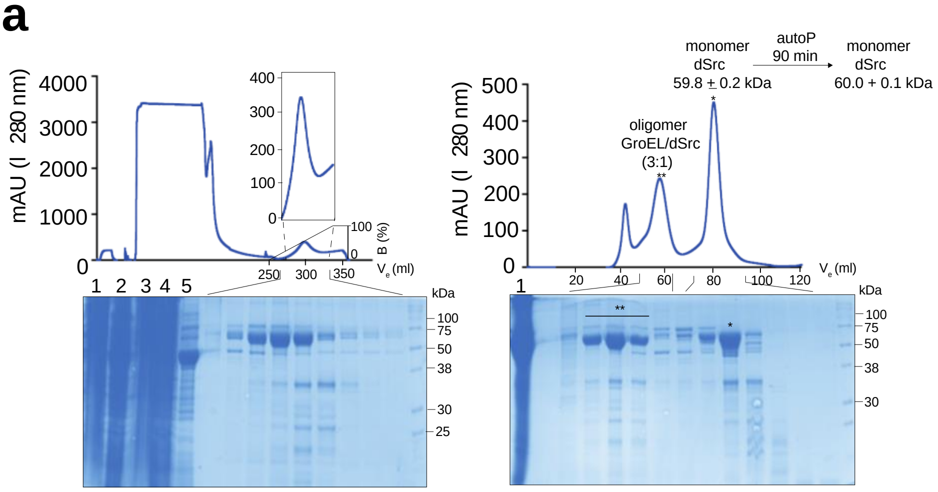

b

Protein IDs	Peptide counts (unique)	Protein names	Gene names	Fasta headers	Number of proteins	Sequence coverage [%]	Mol. weight [kDa]	Intensity
P0A6F5	98	60 kDa chaperonin	groL	sp|P0A6F:	1	99.3	57.328	3.1E+12
Q9V9J3	85	Tyrosine-protein kinase Src42A	Src42A	sp|Q9V9J:	1	96.9	59.069	1.32E+12

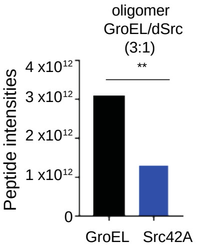

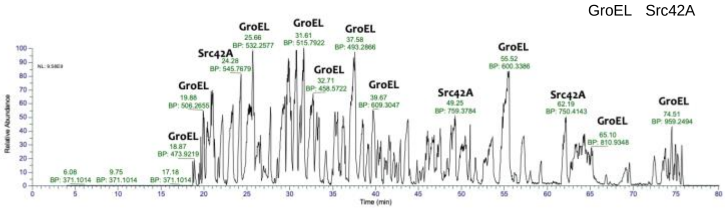

c

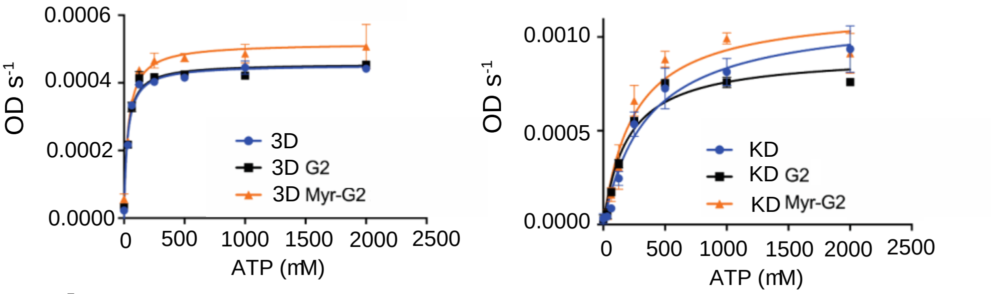

d

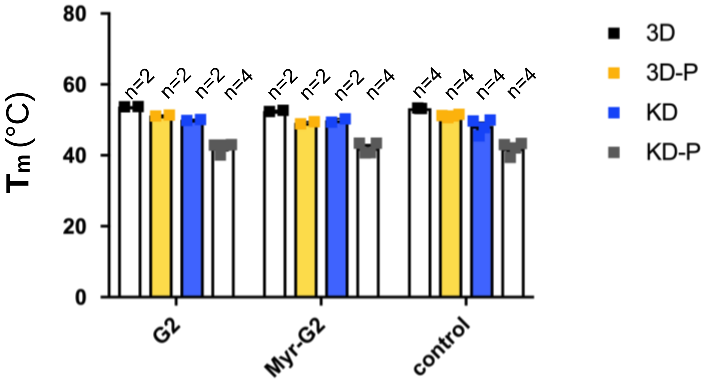

Supplementary figure 1. Purification and characterization of Src42A species by SEC-MALS and MS and functional characterization of Myr-G2 derived peptides.

a) IMAC chromatogram using a HisTrap column (5 ml). Indicated fractions were run on an SDS-PAGE and stained with Coomassie: 1 crude lysate, 2 insoluble fraction, 3 clear lysate, 4 wash, 5 flow-through, left panel. SEC chromatogram using a Superdex 200 16/60 column. Indicated fractions were run on an SDS-PAGE and stained with Coomassie, right panel.

b) Base peak chromatogram (BPC) from LC-MS/MS analysis showing stochiometric relationship of GroEL and dSrc (Src42A) in the oligomeric fraction (**) obtained from the SEC. Data show is from one single experiment, n=1.

c) Enzymatic assays performed with c-Src 3D or KD (both at 1 μM) in apo estate (0-P) or phosphorylated (P, 90 min) incubated for 45 min with a 2.5 molar excess of G2 (GSNKSKPKDASQRRR) and Myr-G2 (Myr-GSNKSKPKDASQRRR) peptides using c-Abl peptide (4mg/ml) as a phosphorylable susbtrate. Data shown are the mean ± SEM of one experiment in duplicate, n=2.

d) DSF data represented in a column plot showing the melting temperature (Tm, °C) of c-Src 3D and KD, both unphosphorylated (0-P) and phosphorylated (P, 90 min) species in apo (control), Myr-G2 and G2 pre-incubated samples. Data represented are the mean ± SEM of 2 experiments, G2/3D (n=2), G2/3D-P (n=2), G2/KD (n=2), G2/KD-P (n=4), , Myr-G2/3D (n=2), Myr-G2/3D-P (n=2), Myr-G2/KD (n=2), Myr-G2/KD-P (n=4), control/3D (n=4), control/3D-P (n=4), control/KD (n=4), control/KD-P (n=4).

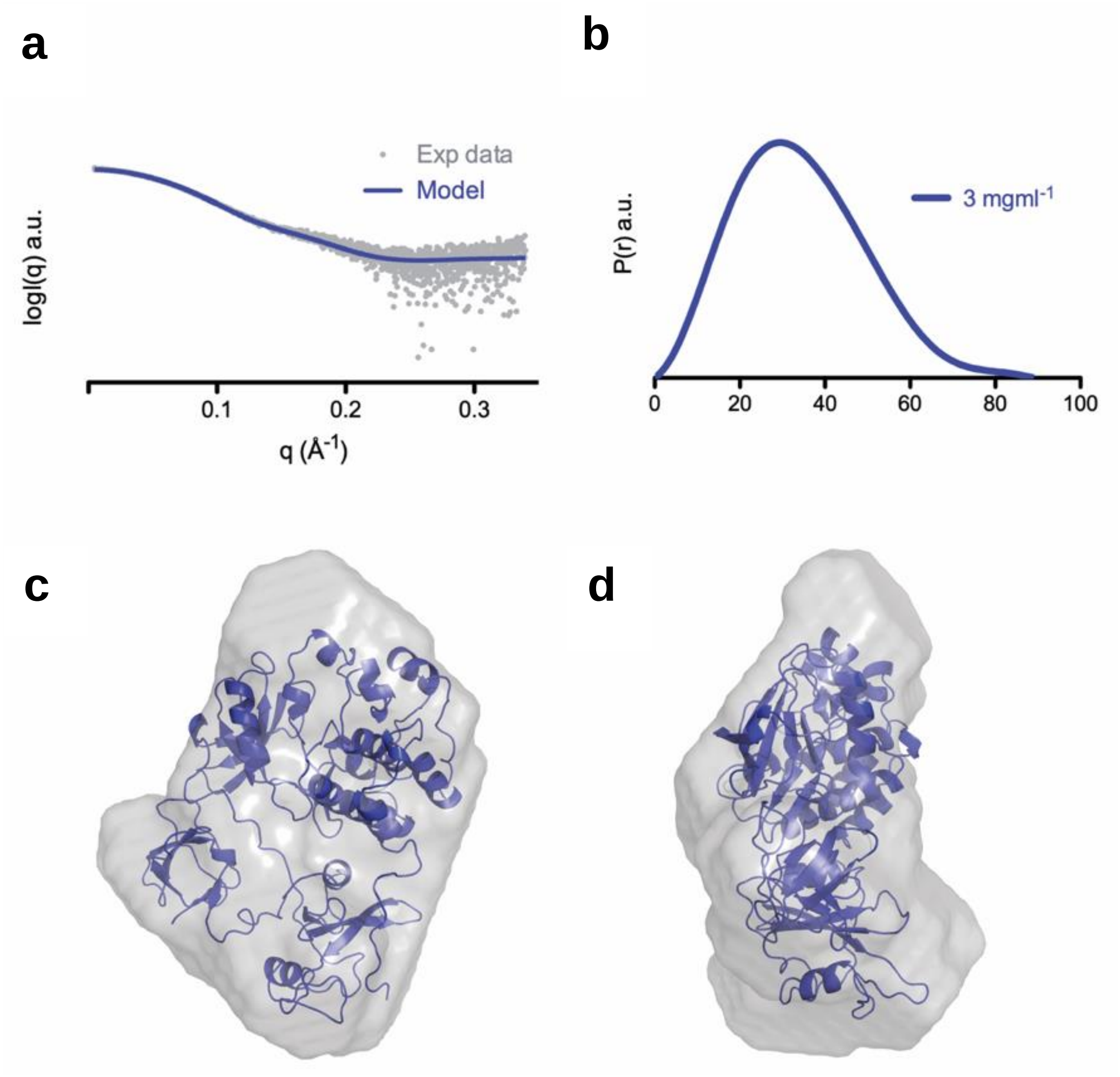

Supplementary figure 2. Analysis by SAXS of Src WT domain organization in solution.

a) Experimental scattering curve (grey dots) and theoretical scattering curve, in blue, computed for the model (smooth), where Src WT adopts a closed conformation as found in the PDB: 2SRC.

b) Normalized pair-distance distribution function P(r) for Src WT (blue graph). The data were offset vertically for clarity, a.u., arbitrary units.

c and d) Overlaying of the ab initio determined SAXS envelope for Src-WT (in pale grey), with the model based on the crystal structure (PDB ID: 2SRC), in blue.

GVTTFVALY_{93}DY_{95}ESRTETDLSFKKGERLQIVNNT_{117}EGDWWLAHSL$S_{128}$$T_{129}GQT_{132}$
GY_{139}IPSNY_{139}VAPSDSIQAEEWY_{152}FGKITRRESERLLLNAENPRGT_{174}FLVRESET_{182}T
KGAY_{187}CLSVSDFDNAKGLNVKHYKIRKLDSGGFY_{216}ITSRTQFNSLQQLVA$Y_{232}$$Y_{233}$SK
HADGLCHRLTTVCPTSKPQTQGLAKDAWEIPRESLRLEVKLGQGCFGEVWMGT_{288}W
NGTTRVAIKTLKPGTMSPEAFLQEAQVMKKLRHEKLVQLYAVVSEEPIY_{338}IVTEY_{343}M
SKGSLLDFLKGETGKYLRLPQLVDMAAQIASGMAY_{379}VERMNY_{385}VHRDLRAANILVG
ENLVCKVADFGLARLIEDNEY_{419}TARQGAKFPIKWTAPEAALY_{439}GRFTIKSDVWSFGIL
LTELTTKGRVPY_{466}PGMVNREVLDQVERGY_{482}RMPCPPECPESLHDLMCQCWRKEP
EERPTFEYLQAFLEDYFTSTEPQY_{530}QPGENL

Residue	Described at biochemical and/or cellular level	Auto-phosphorylation or phospho-site for other kinases	Ref.
Y93	Yes	Phospho-site	[1]
Y95	No	-	
T117	No	-	
S128	No	-	
T129	No	-	
T132	No	-	
Y134	Yes (mouse)	Phospho-site	
Y139	Yes (mouse)	Phospho-site	[2, 3]
Y152	No	-	
T174	No	-	
T182	Yes	Phospho-site	[*]
Y187	Yes	Phospho-site	[4, 5]
Y216	Yes	Phospho-site	[4, 6, 7, 8, 9]
Y232	Yes (rat)	Phospho-site	[**]
Y233	No	-	
T288	No	-	
Y338	Yes	-	[10]
Y343	No	-	
Y379	No	-	
Y385	No	-	
Y419	Yes	AutoP	[11, 12, 13, 14, 15, 16, 17]
Y439	Yes	Phospho-site	[18, 19, 20, 21, 22, 23, 24, 25]
Y466	No	-	
Y482	No	-	
Y530	Yes	Phospho-site	[15, 26, 27, 28, 29, 30]

Supplementary figure 3. Identification of human c-Src phospho sites in vitro by mass spectrometry as described in methods. [*]Ren H (2010) Cell Signaling Technologies (CST) Curation Set: 8825; [**] Hu Y (2009) CST Curation Set: 8467

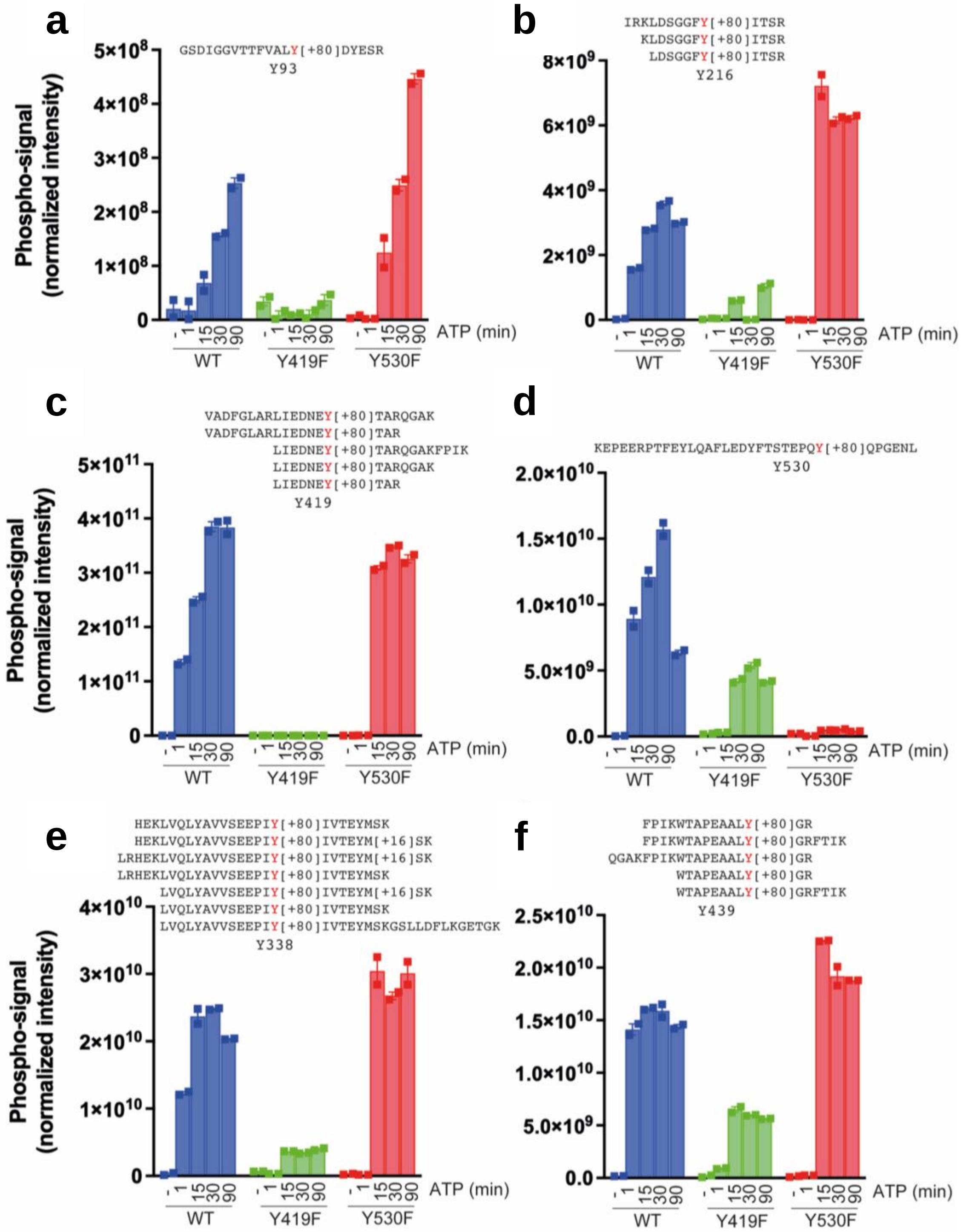

Supplementary figure 4. Identification of human c-Src phospho sites in vitro by mass spectrometry as described in methods. a-f) Time courses (0-90 min) of c-Src 3D WT, Y419F AND Y530F (2 μM). Normalized phospho-peptide signal (mean ± SEM) is representative of one experiment with two technical replicates, n=2. Data is representative of two-independent experiments. All the phospho-peptide sequences used to quantify each phosphorylation site are also shown.

a

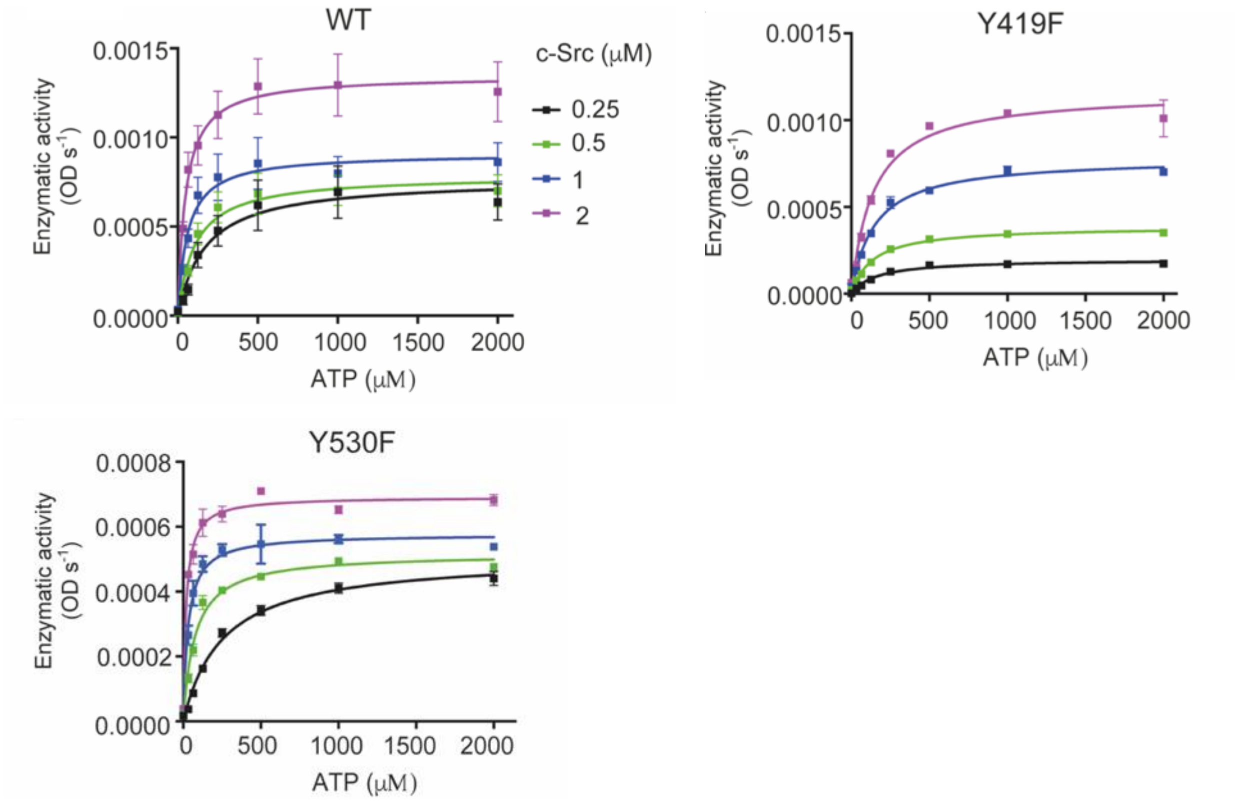

b

**WT**		**0.25 μM**	**0.5 μM**	**1 μM**	**2 μM**
	V_{max} (OD s^{-1})	0.00076	0.00078	0.00091	0.0013
	K_{cat} (s^{-1})	0.48874	0.25080	0.14630	0.1045
	K_M (μM)	170.6	107.1	60.3	48.17
	K_{cat} / K_M (s^{-1} μM^{-1})	0.00286	0.00234	0.00242	0.00216
	ratio	1.0	0.81741	0.84689	0.75725
**Y419F**		**0.25 μM**	**0.5 μM**	**1 μM**	**2 μM**
	V_{max} (OD s^{-1})	0.00019	0.00038	0.00078	0.0016
	K_{cat} (s^{-1})	0.12218	0.12218	0.12540	0.1286
	K_M (μM)	161.5	136.1	147.0	139.7
	K_{cat} / K_M (s^{-1} μM^{-1})	0.00075	0.00089	0.000853	0.00092
	ratio	1.0	1.18	1.12	1.21
**Y530F**		**0.25 μM**	**0.5 μM**	**1 μM**	**2 μM**
	V_{max} (OD s^{-1})	0.0005	0.00051	0.00057	0.0069
	K_{cat} (s^{-1})	0.3215	0.16398	0.091	0.5546
	K_M (μM)	255.4	74.3	31.3	18.3
	K_{cat} / K_M (s^{-1} μM^{-1})	0.0012	0.00220	0.00292	0.0303
	ratio	1.0	1.75308	2.325527157	24.074

Supplementary figure 5. Enzyme kinetics characterization of c-Src (K_M ATP) at increasing enzyme concentrations. a) Enzyme kinetics characterization for ATP at increasing concentrations of enzyme c-Src (3D) WT, Y419F and Y530F. Data shown are the mean ± SEM of the enzymatic activity (OD s^{-1}) for: WT (three experiments in duplicate, n=6), Y530F (two experiments, n=4) and Y419F (one experiment, n=2). b) Table with kinetic parameters and enzymatic constants with fold-changes (ratios) are depicted.

a

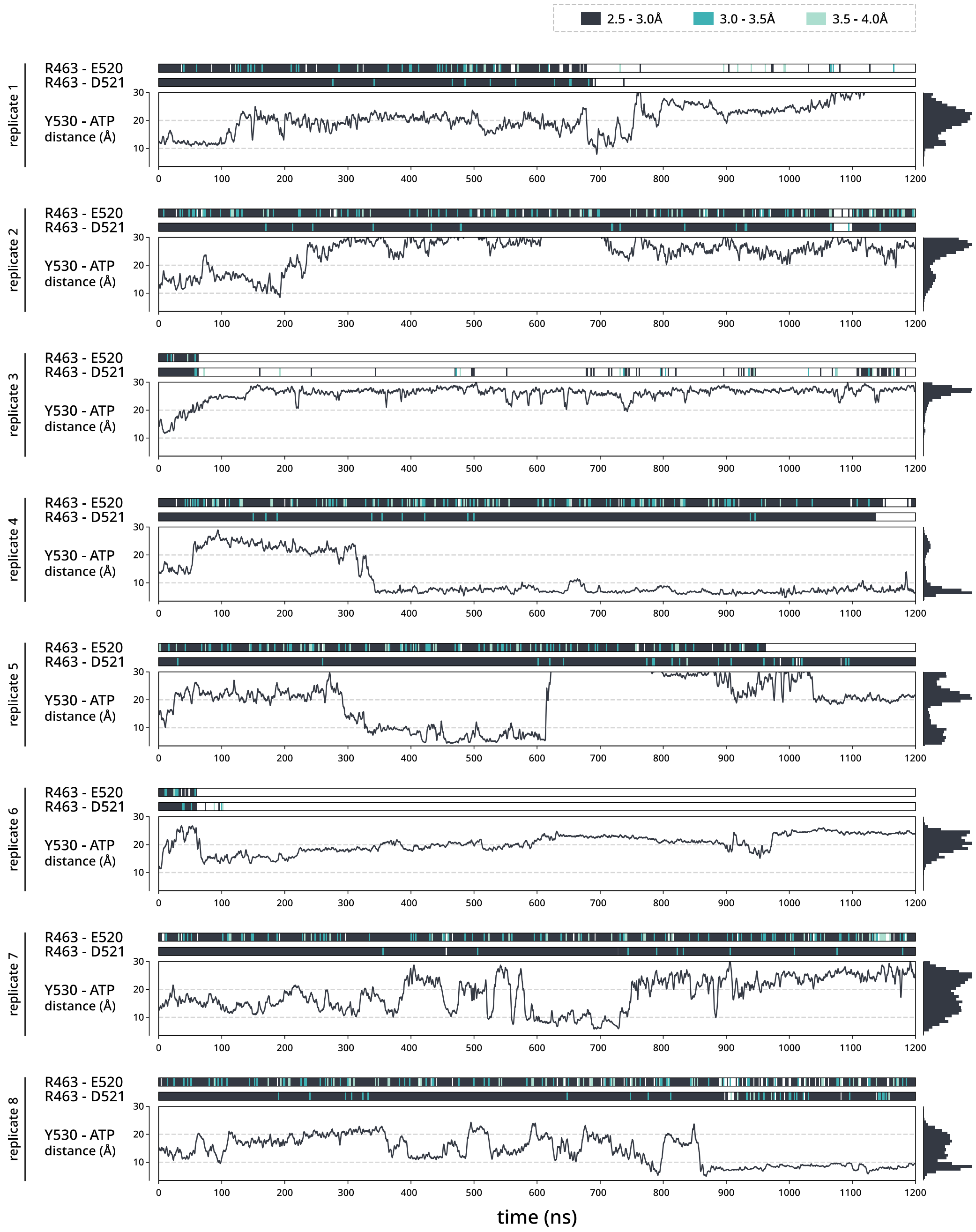

Supplementary figure 6. Molecular dynamic (MD) simulations of an c-Src asymmetric dimer. a) Time-course plots show molecular interactions across eight replicate MD simulations as indicated on the far left. Each replicate includes three time-course plots where the top two show the minimum distance between R463 to E520 (top) and between R463 to D521 (middle). Distance is represented by color as indicated by the legend in the top-right. The line graph (bottom) shows the minimum distance between Y530 and ATP. Distance is represented across the y-axis. The histogram to the right of the line graph shows the distribution of these distances. Simulation time is represented by the shared x-axis at the bottom.

b

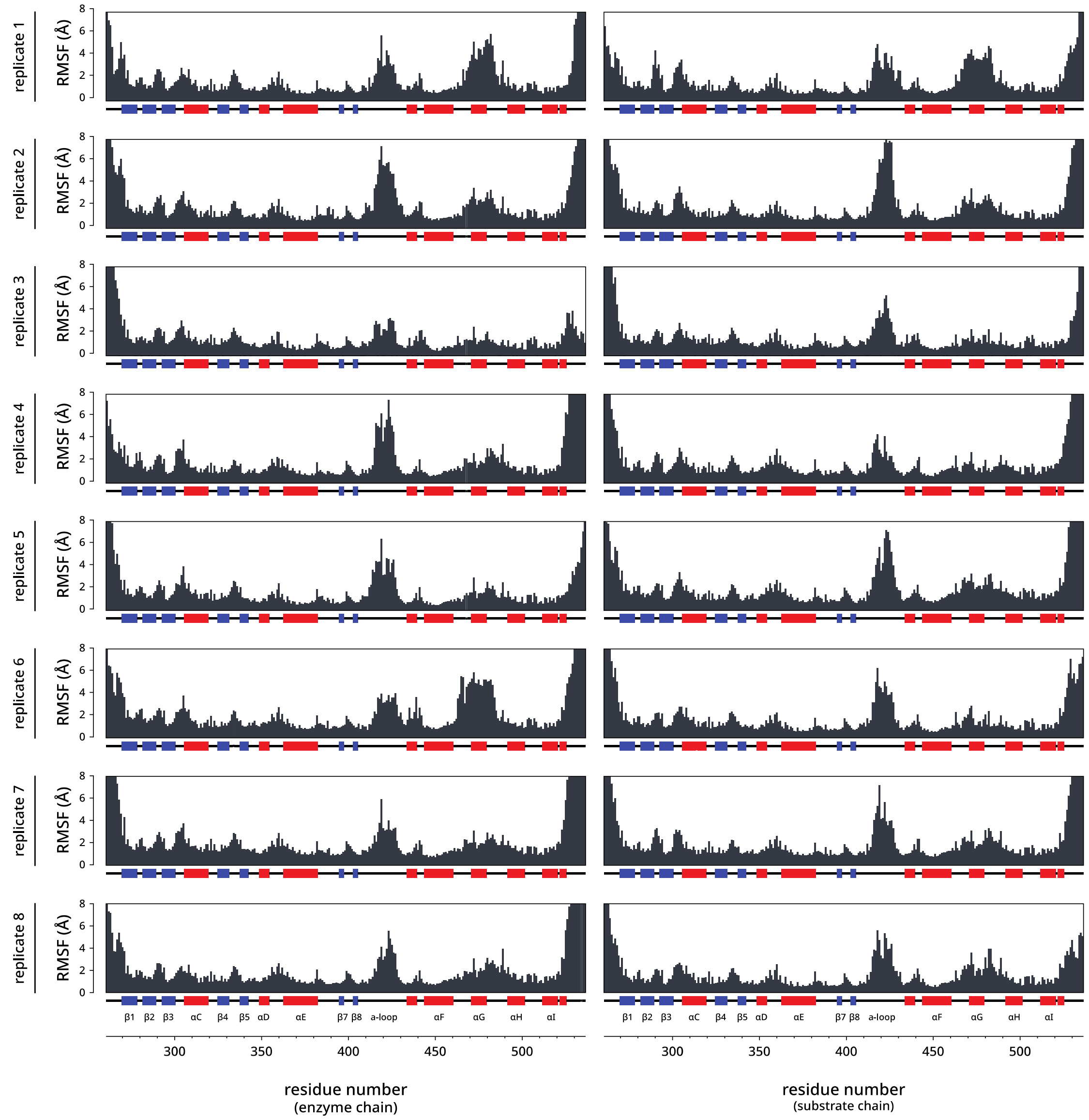

Supplementary figure 6 (continuation). b) We show the root-mean-square fluctuation (RMSF) of each residue across eight replicate MD simulations as indicated on the far left. Within each bar graph, RMSF is indicated on the y-axis, while residue number is indicated on the x-axis. Secondary structure elements are also shown across the x-axis where beta sheets are in blue and alpha helices are in red. RMSF was independently calculated for each enzyme molecule (left side) and the substrate molecule (right side).

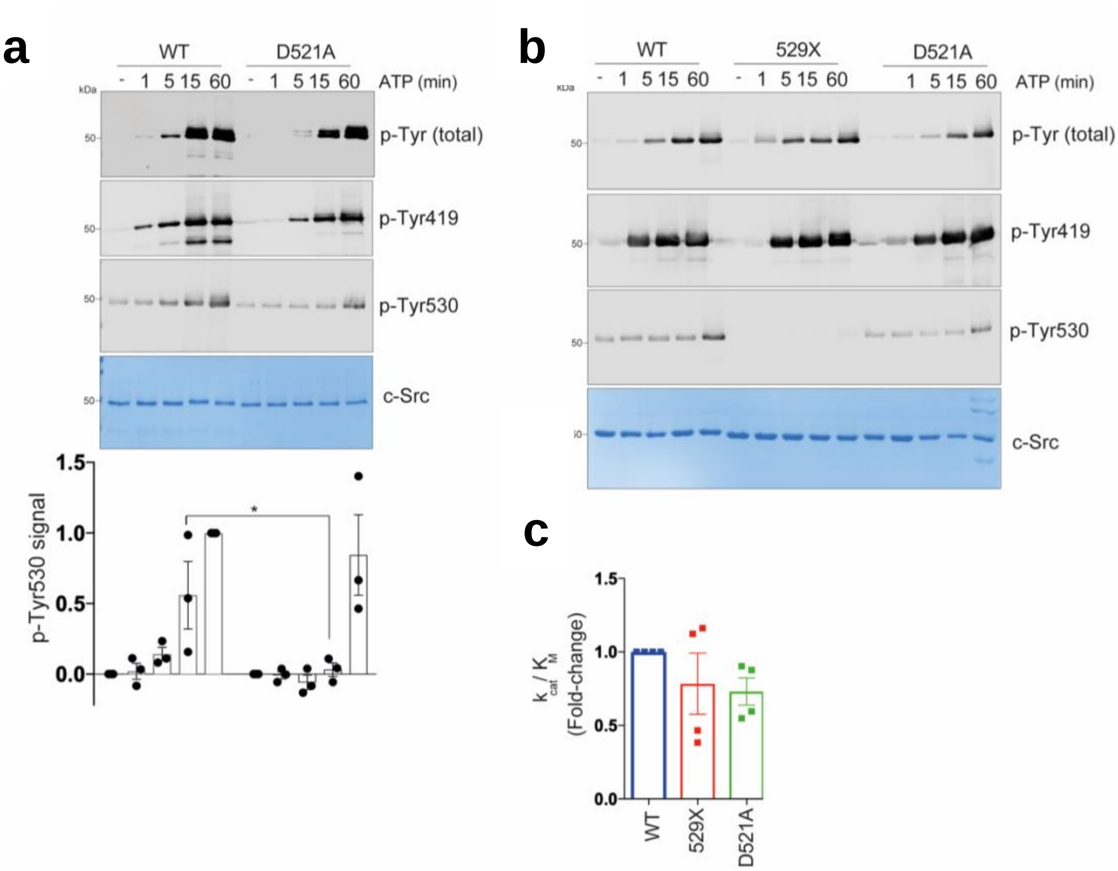

Supplementary figure 7. Functional evaluation of c-terminal D521A and 529X c-Src mutants.

a) WB of samples from a time-course auto-phosphorylation experiment with c-Src WT and D521A (3D-construct 2 μM) in the presence of ATP (1 mM) and $MgCl_2$ (2 mM) for 0–60 min using total and site-specific phospho-tyrosine antibodies. Total c-Src protein was evaluated by Coomassie staining. Quantification of the phospho-Tyr 530 signal, data represent the mean ± SEM of 3 independent experiments (n=3). Multiple comparison one-way ANOVA test, * p = 0.0399.

b) WB of samples from a time-course auto-phosphorylation experiment with c-Src WT, 529X and D521A (3D-construct 2 μM) in the presence of ATP (1 mM) and $MgCl_2$ (2 mM) for 0–60 min using total and site-specific phospho-tyrosine antibodies. Total c-Src protein was evaluated by Coomassie staining. Data are representative of 3 independent experiments

c) Enzyme kinetics characterization showing catalytic efficiency constant (k_{cat}/K_M ATP) of c-Src (3D-construct 1 μM) WT and indicated mutants. Data represent the fold-change vs control (WT) of the mean ± SEM of two independent experiments in duplicate, n=4.

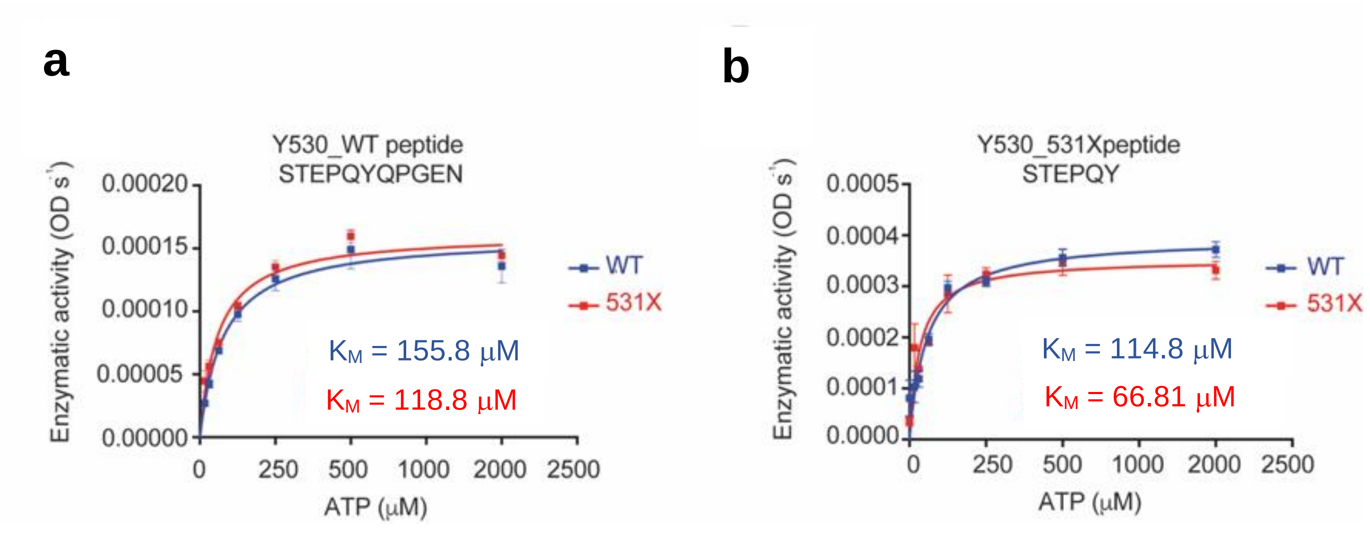

Supplementary figure 8. Enzyme kinetics of c-Src WT and 531X using c-terminal derived peptides. Enzyme kinetics characterization of c-Src (3D-constructs, 1 μM) WT and 531X mutant using: a) an intact c-terminal peptide containing Y530 (STEPQYQPGEN) and b) a palindrome truncated peptide that mimics 531X c-terminal sequence (STEPQY). K_M values are depicted. Data represented are the mean ± SEM of 2 experiments in duplicate, n=4.

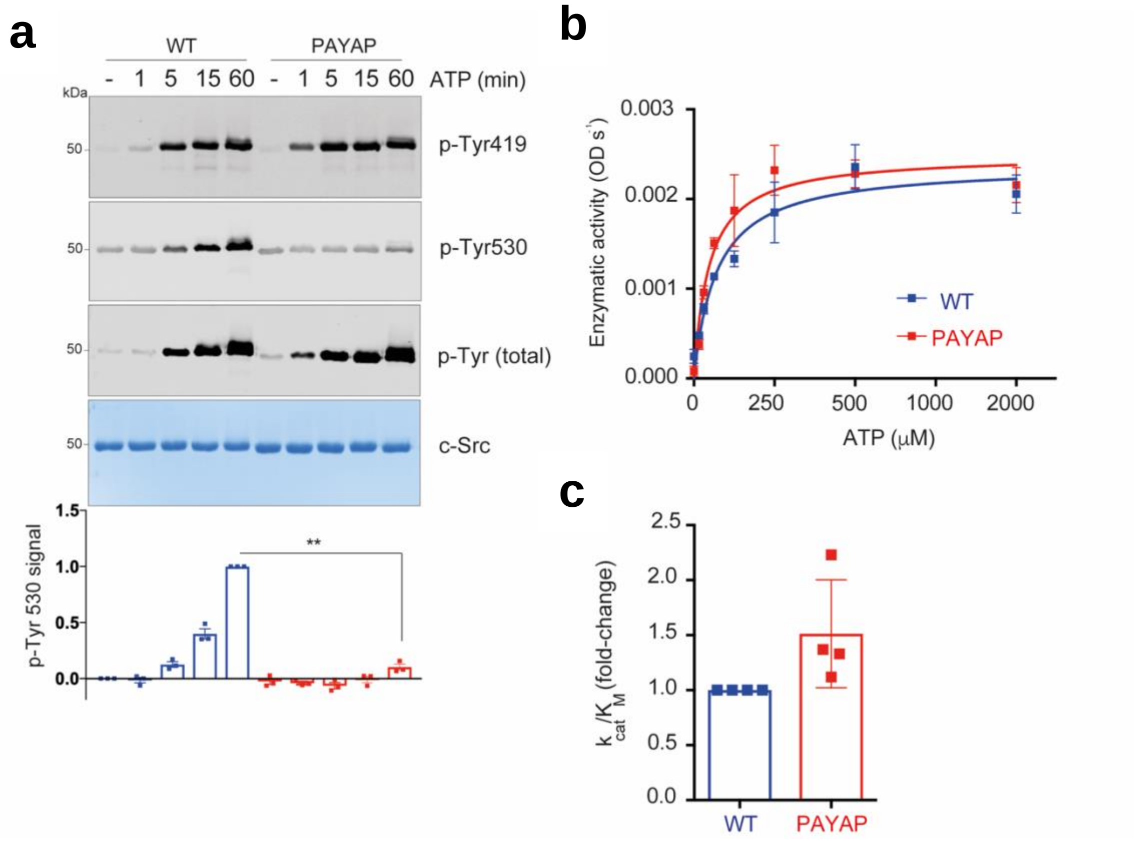

Supplementary figure 9. Functional evaluation of a c-terminal palindrome PAYAP mutant.

a) WB of samples from a time-course auto-phosphorylation experiment with c-Src WT and a PAYAP mutant (3D-constructs, 2 μM) in the presence of ATP (1 mM) and $MgCl_2$ (2 mM) for 0–60 min using total and site-specific phospho-tyrosine antibodies. Total c-Src protein was monitored by Coomassie staining. Quantification of the phospho-Tyr 530 signal, data represent the mean and SEM of 3 independent experiments (n=3). Multiple comparison one-way ANOVA, ** p = 0.0033.

b) Enzyme kinetics of c-Src WT and PAYAP mutant (3D-constructs, 1 μM) using an ABL derived peptide (EAIYAAPFAKKK) at increasing concentrations of ATP. Data represented are the mean ± SEM of two experiments performed in duplicate (n=4), upper panel. Catalytic efficiency constant (k_{cat}/K_M, fold-change versus control WT) of the experiment from a), lower panel.

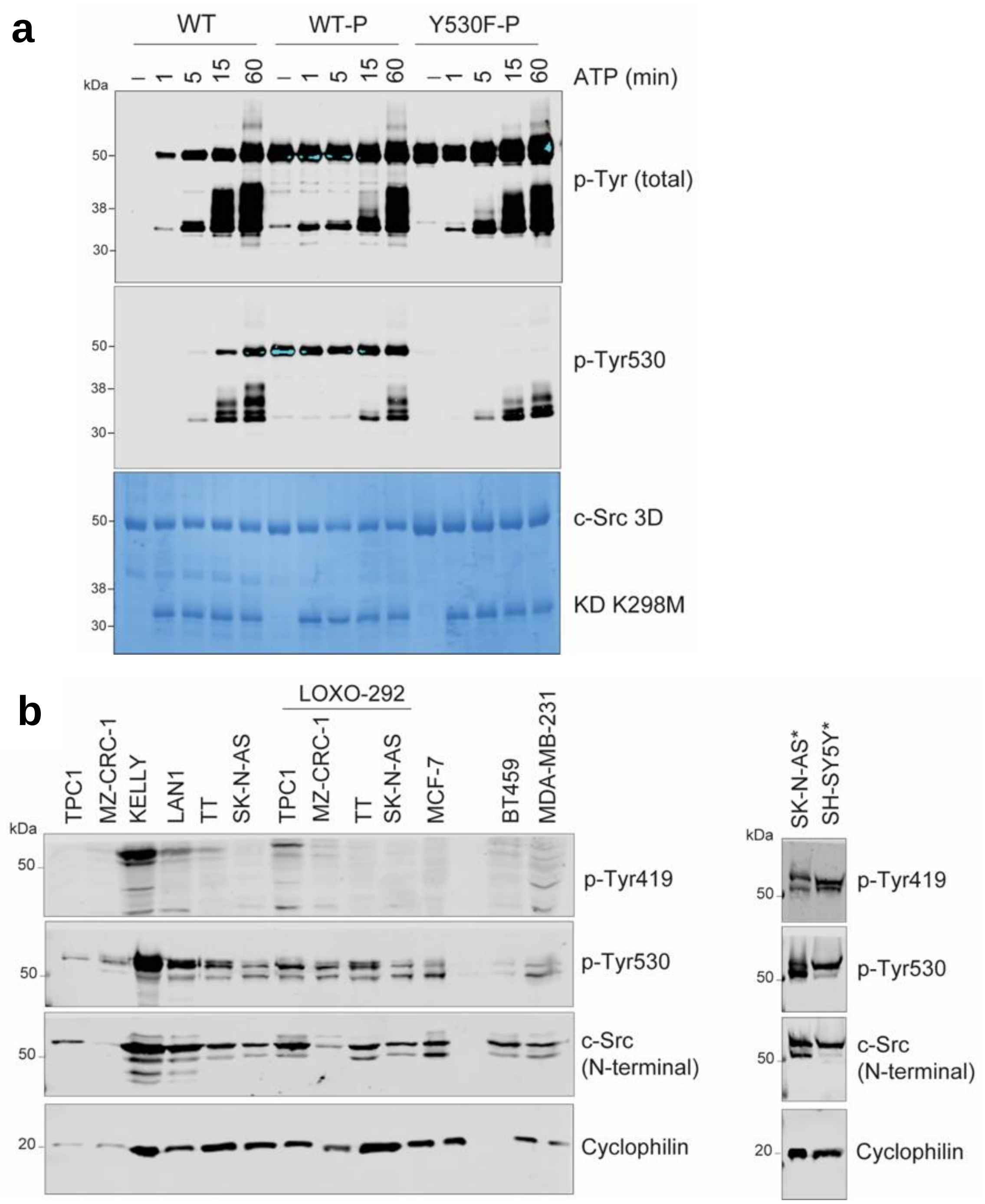

Supplementary figure 10. a) WB analyses of a phosphorylation experiment with c-Src WT, already phosphorylated (90 min) WT and Y530F (3D constructs, 1.5 μM) versus a RET KD K298M (3 μM) as a substrate surrogate using the indicated antibodies. Total protein levels were visualized with Coomassie staining. b) WBs experiments of whole cell lysates from the indicated cancer cell lines using the indicated antibodies, (*) cells grown in the presence of non-essential amino acids. First antibodies dilutions 1:1K.

2. SUPPLEMENTARY TABLES

Supplementary table 1. SAS sample, data collection, analysis and 3D modelling details [31] for c-Src in solution.

Scattering particle composition	
Protein	P12931
Sample environment/configuration	
Solvent composition	20 mM Tris pH 8.0, 150 mM NaCl, 1% glycerol, 1 mM DTT
Sample temperature (°C)	4
In beam sample cell (ºC)	18
Size Exclusion Chromatography SEC-SAS	
Sample injection concentration, (mg/ml)	6
Sample injection volume, mL	40
SEC column type	Superdex 200 Increase 3.2 column (Cytiva)
SEC flowrate, mL/min	0,16
Data acquisition/reduction software	GDA/Scåtter
Source/instrument description or reference	B21, Diamond Light Source
Measured q-range (q_{min} – q_{max}; Å^{-1})	0.0031-0.38
Method for scaling intensities	Relative to water (0.0163 Å^{-1})
Exposure time (s) and final number of sample frames	3, 599
Relevant details	
Methods/Software	PRIMUS, AUTORG, GNOM
Guinier Analysis	
I (0) ± σ (cm^{-1})	0.0494563
R_g ± σ (Å)	26.14±0.021
Data point range	69-347
Linear fit assessment	99
PDDF/P(r) analysis	
I (0) ± σ (cm^{-1})	0.04936
R_g ± σ (Å)	26.10±0.05
d_{max} (Å)	89
q-range (Å^{-1})	0.0032-0.38
P(r) fit assessment	0.004762-0,339869
Porod volume, V_p (Å^3)	674

Molecular weight (M) estimates (kDa)	
From chemical composition	53
From SAS, concentration independent method (MoW)	52,7
From SAS-independent measure (MALLS)	52,3
Shape modelling method	
Software	FoXS
q-range for fit ($q_{min} – q_{max}$; Å^{-1})	0,0045-0,3397
Symmetry/anisotropy assumptions	N/A
Number of individual model reconstructions	1 (PDB: 2SRC)
χ^2, CorMap P-values for fit	2,97, N/A
Atomistic modelling methods	
Software	DAMMIN, DAMMIF, FoXS
q-range for fit ($q_{min} – q_{max}$; Å^{-1})	0,0045-0,3397
Symmetry/anisotropy assumptions	P1
Number of individual model reconstructions	20
χ^2, CorMap P-values for fit	2,41, 0,18
Data and model deposition	
SASBDB IDs	SASDSV8

3. SUPPLEMENTARY METHODS

Extended methods

Plasmids

A pET28a-TEV plasmid codifying an N-Terminal 6xHis tag followed by a Tobacco etch virus (TEV) protease recognition site was used to express human c-Src (UniProtKB P12931) three domain (3D, SH3-SH2-KD aa 84-536) or kinase domain (KD, aa 254-536) codifying sequences. Both pFCDUET-YopH phosphatase and pGKJE8-GroEl/GroEs chaperones plasmids (Takara chaperone plasmid set cat. # 3340) were used for co-expression experiments. These constructs were a kind gift of Dr. Daniel Lietha (CIB, Madrid). For expression of recombinant drosophila c-Src isoform 42A (Src42A, UniProtKB Q9V9J3) we used a pET-His10-Src42A plasmid (Addgene #126674) codifying a full-length drosophila isoform (aa 1-517) with a N-terminal 10x His tag and a Thrombin protease recognition site. For experiments in Drosophila a pUAST-attB plasmid encoding drosophila Src homolog isoform A (Src42A) was cloned by amplifying dSrc42a from a pGEX-Src42a donor plasmid (Addgene #126673) using primers with EcoRI (forward 5´- CTGAATAGGGAATTGGGAATTCATGGGTAACTGCCTCACC-3´) and XbaI (reverse 5´- CCTTCACAAAGATCCTCTAGATCAGTAGGCCTGCGCCTC-3´) restriction sites.

Site-directed mutagenesis

Site-directed mutagenesis was performed in order to generate all the point mutants and variants described in this study using the Q5-site directed mutagenesis kit (New England Biolabs) following manufacturer instructions and the indicated primers.

K298M-forward	5´- GGTGGCCATCATGACCCTGAAGC - 3´
K298M-reverse	5´- CTGGTGGTACCGTTCCAG - 3´

Y419F-forward 5´- AGACAATGAGTTCACGGCGCGGC - 3´

Y419F-reverse 5´- TCAATGAGCCGAGCCAGC - 3´

Y530F-forward 5´- CGAGCCCCAGTTCCAGCCCGGGG - 3´

Y530F-reverse 5´- GTGGACGTGAAGTAGTCCTCCAGGAAGG - 3´

dY400F-forward 5´- GGAGGACGAATTCGAGGCGCGGG - 3´

dY400F-reverse 5´- TTGATGAGCCTAGCTAAACCAAAGTCG -3´

P528X/Q529X-forward 5´- GTCCACCGAGTAATAGTACCAGCCCG -3´

P528X/Q529X-reverse 5´- GTGAAGTAGTCCTCCAGG -3´

Q531X/P532X-forward 5´- GCCCCAGTACTAGTAGGGGGAGAACC -3´

Q531X/P532X-reverse 5´- TCGGTGGACGTGAAGTAG -3´

D521A-forward 5´- CTTCCTGGAGGCCTACTTCACGTC -3´

D521A-reverse 5´- GCCTGCAGGTACTCGAAG -3´

PAYAP-forward 5´- CGCGCCCGGGGAGAACCTCTAG -3´

PAYAP-reverse 5´ - TACGCGGGCTCGGTGGACGTGAA -3´

Briefly, in an initial exponential amplification step template plasmid DNA (1-25 ng) was incubated with Q5 Hot Start High-Fidelity master mix (2X), 10 µM of forward and reverse primer stock to a final concentration of 0.5 µM and nuclease-free water in a final volume of 12.5 µl. Amplification and cycling conditions were as followed: i) initial denaturation at 98°C 30 sec, ii) 98 °C 10 sec, 50–72 °C 30 sec, 72 °C 4 min (25 cycles) and iii) final extension 72 °C (2 min). For mutagenic primers, we used the T_a provided by the online NEB primer design software, NEBaseChanger™. Next, a kinase-ligase and Dpn-I (KLD) reaction was set at room temperature for 5-30 min, in which a 1 µl of PCR product was incubated with a 2X KLD reaction buffer, 10X KLD enzyme mix and nuclease-free water in a total volume of 5 µl. Q-5 site directed mutagenesis products (5 µl) were transformed

in E. coli Q5-DH5α by incubation for 30 min on ice and heat shocked at 42 ºC for 45 sec after which samples were placed back on ice for 2-3 min prior recovery in 500 μl of antibiotic free LB media at 37 ºC and 200 rpm shaking for one hour. Next, 150 μl of recovered culture was plated onto LB-Agar plates with kanamycin (50 mg/ml) and left overnight (o/n) into an incubator at 37 ºC. Next day, one single colony was amplified o/n in 10 ml of LB media with kanamycin (50 μg/ml) and the next morning the bacterial culture was pelleted by centrifugation at 1.600 x g in a eppendorf centrifuge 5810 R and plasmid DNA purification was performed using the E.Z.N.A. plasmid DNA mini kit (Omega Bio-Tek Inc) following manufacturer instructions. Briefly, bacterial pellet was resuspended in 250 µl Solution I (resuspension solution) supplemented with RNAse A (1/1000 v/v). Then, the same volume of Solution II (lysis solution) was added, mix and incubated for 2 min. Then, 350 µl solution III (neutralization solution) was added and centrifuged at 16.100 x g in an eppendorf centrifuge 5415 D for 10 min at room temperature. Supernatant was transferred to a HiBind® DNA mini column and centrifuged for 1 min and flow through discarded. Then, 500 µl HBC solution was added, centrifugated for 1 min and flow through discarded again. Process was repeated with 700 µl of DNA washing buffer. Then, 50 µl of elution buffer was added and centrifuged for another minute on a clean eppendorf. Plasmid concentration and purity was checked by absorbance using a nanodrop (Thermo Scientific NanoDrop One). All mutagenesis products were confirmed by DNA Sanger sequencing at the CNIO Genomics Unit.

Expression and purification of recombinant proteins

In order to express high yields of soluble, un-phosphorylated and monodisperse recombinant human c-Src protein we followed a modified protocol [32] in which in addition to YopH phosphatase, we also co-expressed the chaperone GroEl. For co-expression, pET28a-TEV-hSrc [84-536] and pET28a-TEV-hSrc [254-536] plasmids were co-transformed in E. coli BL21 bacteria strand previously transformed with pFCDUET-YopH and pGKJE8-GroEl/GroEs plasmids and grown in 50 ml of LB media containing

kanamycin (50 μg/ml), streptomycin (50 μg/ml) and chloramphenicol (34.5 μg/ml) overnight at 37ºC shaking at 200 rpm in a 250ml Erlenmeyer flask. Next day, the bacterial culture was diluted (1:100) in LB media with the same antibiotic composition and concentration using Fernbach baffled Erlenmeyer flasks. When the bacterial culture reached an optic density (λ 600 nm) of 0.15, the culture was cooled to 18 ºC and tetracycline was added at a final concentration of 1ng/ml in order to trigger chaperone expression. After approximately one hour and always at an optic density of 0.4-0.5 at λ 600 nm, IPTG was added to a final concentration of 500 μM and culture was left overnight agitating (200 rpm) at 18ºC. To express recombinant dSrc42A we followed the same procedure but with the co-expression of YopH only, so a pET-His10-Src42A plasmid was transformed in BL21 previously transformed with the pCFDUET-YopH construct and tetracycline was not added at any moment.

Next day, bacterial culture was harvested at 1000 x g and 4ºC with a JLA 8.100 rotor in a Beckman Coulter Avanti J-20XP centrifuge. Supernatant was discarded and pellet was transferred into a 50 ml Falcon centrifuge tube and frozen at -20 ºC for later purification. Alternatively, the pellet was immediately resuspended in 50 ml lysis buffer (50 mM Tris pH 8, 500 mM NaCl, 0.1mM PMSF) and sonicated on ice with a Bioblock Scientific Vibra Cell 75042 sonicator with a model CV33 sound radiator for 4 minutes (9 seconds on-3 seconds off, 37% amplitude). Crude lysate was transferred into a JA 25-50 rotor tubes and centrifugated at 48000 x g at 4ºC for 45 min in a Beckman Coulter Avanti J-25 centrifuge. Soluble clarified supernatant underwent a further 10 sec sonication step and was filtered through a 45 μm Jet Biofilter with a syringe.

Human c-Src was purified by three chromatographic steps (see figure 1). First, lysate was passed through an immobilized metal anion chromatography (IMAC) 5 ml column (GE Healthcare HisTrap^{TM} HP) equilibrated with 5 column volumes (CVs) of IMAC buffer A (20 mM Tris pH 8, 150 mM NaCl, 1 mM TCEP, 5% Glycerol) with an GE Healthcare AKTA PURE FPLC at flowrate of 5 ml/min. Next, the column was washed with 90% IMAC

buffer A and 10% IMAC buffer B (20 mM Tris pH 8, 150 mM NaCl, 300 mM Imidazole, 1mM TCEP, 5% Glycerol) until absorbance signal at a λ of 280nm was stable (usually 10 CVs), after which a 100% gradient with IMAC buffer B was run in 100 ml (20 CVs) and 20 fractions of 5 ml were collected. Fractions were tested in a 12% SDS-PAGE gel and Coomassie blue staining.

Fractions expressing recombinant c-Src were pooled together and diluted in IEC (ionic exchange chromatography) buffer A (20 mM Tris pH 8, 1 mM DTT, 5% glycerol) up to 3 times original volume so NaCl concentration was lowered to 50 mM. Then, sample was loaded into an IEC column (GE Healthcare HiTrap Q HP column) previously equilibrated with 5 CVs of IEC buffer A at a flowrate of 5ml/min. After loading the column was washed until absorbance signal at λ 280nm was stable (4-5 CVs) and then a 100% gradient with IEC buffer B (20 mM Tris pH 8, 500 mM NaCl, 1 mM DTT, 5% glycerol) was run in 100 ml (20 CVs) and 20 fractions of 5ml each were taken. Fractions were tested in a 12% SDS-PAGE gel and Coomassie blue staining.

Fractions expressing recombinant c-Src were pulled together and mixed with a His-tagged rTEV protease (20-40 μM) in a 1/20 molar TEV/c-Src stoichiometry. Buffer was supplemented with 2mM TCEP and digested at 4ºC o/n. Alternatively, digestion was performed for 2 hours at room temperature. Next, a His-trap reverse step was undertaken to remove the protease and tag of the recombinant protein. Briefly, His-rTEV protease digested c-Src sample (input) was passed through the HisTrap column previously equilibrated with IMAC buffer A at 2 ml/min flowrate with a peristaltic pump (GE Healthcare Pump P-1) and then washed with 5 CVs of buffer IMAC A supplemented with 50 mM imidazole. Flow through (FT) was taken and check together with the input in a 12% SDS-PAGE gel and Coomassie blue staining. FT was taken and concentrated with a Millipore concentrator with a 10-30 kDa cut-off by centrifugation with an Eppendorf Centrifuge 5810 R at 2.178 g at 4 ºC. When c-Src volume was less than 2.5 ml it was injected in a 5ml loop (GE Healthcare) to run a size exclusion chromatography (SEC)

with a Superdex 200 16/60 column (GE Healthcare) previously equilibrated with 1.5 CVs of SEC buffer (20 mM Tris pH, 150 mM NaCl, 1 mM DTT, 5% Glycerol). Fractions of 5 ml were collected and tested in a 12% SDS-PAGE gel and Coomassie blue staining. Protein concentration and purity were checked by absorbance using a nanodrop.

In the case of dSrc 42A purification, only two chromatography steps were undertaken: First, clarified lysate was passed through an IMAC 5 ml column (GE Healthcare HisTrap HP) equilibrated with 5 CVs of buffer A (20 mM Tris pH 8, 150 mM NaCl, 1 mM TCEP, 5% Glycerol) with an GE Healthcare AKTA PURE FPLC at a flowrate of 5 ml/min. After loading the column was washed with 90% buffer A and 10% buffer B (20 mM Tris pH 8,150 mM NaCl, 500 mM Imidazole, 1 mM TCEP, 5% glycerol) until absorbance signal at λ 280nm wavelength signal was stable; typically, it was for 7 CVs. Then, a 100% gradient of buffer B was run in 100 ml 20 CVs taking 20 fractions of 5ml each. Fractions were tested in a 12% SDS-PAGE gel and Coomassie blue staining. Fractions expressing recombinant dSrc42A were pulled together and concentrated in a Millipore concentration (30 kDa cut-off) as indicated before. Concentrated sample (2.5 ml) was injected using a 5 ml loop in an AKTA PURE FPLC (GE Healthcare) to run a SEC with a Superdex 200 16/60 column (GE Healthcare). Fractions were tested in a 12% SDS-PAGE gel and Coomassie blue staining. Protein concentration and purity were checked by absorbance using a nanodrop.

Size-Exclusion Chromatography with Multi-Angle Light Scattering (SEC-MALS)

A sample volume of 400-500 μl at a minimum concentration of 100 μg was injected in a Superdex 200 Increase 10/300 column (Cytiva) equilibrated in 20 mM Tris pH, 150 mM NaCl, 1mM DTT, 5% Glycerol buffer (filtered through a 0.1 μm filter) and connected to an AKTA Purifier equipment (GE Healthcare). The chromatographic eluent was monitored by three consecutive detectors in series: (1) a multi-wavelength UV-Vis absorbance detector Monitor UV-900 of the AKTA system (GE Healthcare) with a 10 mm path length flow cell, (2) a light scattering DAWN Heleos 8+ (Wyatt Technology) with

detectors at eight different angles (from 32 to 141 degrees from the source) using a linearly polarized GaAs laser operating at 665 nm and (3) an Optilab T-rEX (Wyatt Technology) differential refractive index detector with a laser wavelength of 658 nm. The column was equilibrated overnight in running buffer at 0.1 ml/min flow to obtain stable base lines for the detectors before data collection. After that, all the experiments were performed at 0.5 ml/min flow and room temperature (~25 ºC). Before running test samples, a control run with BSA (500 μl at 0.5 mg/ml), a well-characterized monodisperse sample, was carried out to set the alignment and band broadening parameters and the normalization coefficients of the MALS detectors necessary for data analysis. Data collection and analysis were performed using UNICORN 5.10 (GE Healthcare) and ASTRA 6.0.3 (Wyatt Technology) software packages.

Protein electrophoresis

Protein electrophoresis were performed with 12% SDS-PAGE gels. The separating gel polymerized with 375 mM Tris pH 8.8, 12% acrylamide/Bis 30% w/v, 0.1% SDS, 0.1% ammonium persulfate (APS) and 0.5% TEMED. Gel staking polymerized with 125 mM Tris-Cl pH 6.8, 12 % acrylamide, SDS 0.1%, APS 0.1% and TEMED 1%. These gels were run in electrophoresis buffer (25 mM Tris pH 8.3, 192 mM glycine, 0.1% SDS) using the Mini-PROTEAN system (BIO RAD) at a constant 200mV for 30 min using a BIO RAD PowerPac Basic. For Coomassie staining, gel was immersed in Coomassie blue staining solution (10% acetic acid, 40% absolute ethanol and 50% deionized water with 1g/l Brilliant blue 250 R. Once the gel was blue (typically, after 30 min) it was immersed in Coomassie distaining solution (10% acetic acid, 50% absolute ethanol, 40% deionized water).

Western blotting and antibodies

SDS-PAGE gels were transferred onto nitrocellulose 0.2 μm (Amersham™) or PVDF 0.45 μm (Millipore) membranes using the Mini-PROTEAN system (BIO RAD). PVDF

membranes were activated first in ethanol 100% for 10 min. SDS-PAGE gel and membrane were sandwiched between four filter paper slices in mini-PROTEAN system immersed in transfer buffer (25 mM Tris pH 8.8, Glycine 190 mM and ethanol 10%) and run at constant 200 mV for 120 min on ice. Transferred membranes were immersed in blocking solution (10 mM Tris pH 8, 150 mM NaCl, 5% weight/volume (w/v) skimmed powder milk) for 60 min shaking at 15 rpm on a shaker see-saw rocker SSL4 (STUART). After blocking membranes were washed three times with TBS-T (10 mM Tris pH 8, 150 mM NaCl, Tween-20 0.1% v/v) prior incubation with primary antibody solution (TBS-T with BSA 5% w/v) o/n at 4ºC shaking at 15 rpm on a Duomax 1030 (Heidolph) shaker. Antibodies used were phospho-Src Tyr419 (D49G4) CST #6943 and Src (36D10) CST #2109 were diluted at 1/10000 and antibodies phospho-Src Tyr 530 (ThermoFisher 44-662G and CST#2105), total phospho-Tyr (p-Tyr-100 CST #9411) at 1:5000, total Src N-terminal 32G6 rabbit monoclonal (CST #2123, 1:1000), and Cyclophilin (D1V5J) rabbit monoclonal antibody (CST #43603, 1: 1000). Next day membranes were washed with 20 ml TBS-T 3-to-5 times and immersed in secondary antibody solution (TBS-T skimmed powder milk 5% m/v). Secondary antibodies anti-rabbit or -mouse IgG DyLight conjugate at 680 or 800 nm (CST #5366 #5151 #5470 #5257) were used at half the dilution factor o the primary for one hour at room temperature protected from light. After incubation with secondary antibodies, membranes were washes 3-to-5 times with TBS-T. Next, membranes were scanned in an Odyssey CLx scanner and images exported.

In vitro phosphorylation assays

In vitro phosphorylation assays were performed at room temperature using recombinant human c-Src 3D and KD (WT and indicated mutants) and full-length dSrc42A at 1-2 µM final concentration in buffer (20 mM Tris pH, 150 mM NaCl, 1 mM DTT, 5% Glycerol, 2 mM $MgCl_2$) and ATP (1 mM) at the indicated time points (unstimulated, 1, 5, 15, 30, 60 and 90 min). Aliquots from the time-course were mixed with 5X Laemmli sample buffer

(ThermoFisher) and denaturalized at 95 ºC during 1-2 min in a thermoblock AccuBlock (Labnet).

Enzymatic assays

Phosphorylation rates of peptide substrates by recombinant c-Src and variants at 1μM final concentration were determined by using an NADH-coupled pyruvate kinase assay in presence of increasing ATP concentrations. The enzyme-substrate solution (20 mM Tris-Cl, 1 mM $MgSO_4$, 400 mM Phosphoenolpyruvate (PEP), 100 mM NADH, 2450U/ml Pyruvate kinase (PK), 2.26 mg/ml Lactate dehydrogenase (LDH) was prepared at different concentrations of ATP: 0, 0.08, 0.16, 0.32, 0.65, 1.25, 2.5 and 5 mM. The experiments were performed in a 384 well plate (Greiner bio-one) and the NADH consumption was read at 340 nm wavelength during 120 cycles of 30 sec each by using a Victor multilabel plate reader 1420 Multilabel Counter (Perkin Elmer). In order to obtain catalytic rates and kinetic constants by Michaelis-Menten equation, experiments were analyzed using Prism software. The following peptides sequences were used as exogenous substrates: c-Abl (EAIYAAPFAKKK), c-Src Tyr 419 (IEDNEYTARQG), c-Src Tyr 530 (STEPQYQPGEN), c-Src 531X (STEPQY), c-Src G2 (GSNKSKPKDASQRRR), c-Src Myr (Myr-GSNKSKPKDASQRRR) and RET Tyr 900/905 (DVYEEDSYVK).

Mass Spectrometry

In-gel digestion: excised SDS-PAGE bands were washed in 50 mM NaHCO3/ Acetonitrile (50/50, v/v) and proteins digested using the standard procedure. Proteins were reduced (15 mM TCEP, 30 min at RT in the dark) and alkylated (30 mM CAA) and subsequently digested with trypsin in 50 mM $NaHCO_3$ overnight at 37 °C (Promega) at an estimated protein:enzyme ratio of 1:100. In-solution digestion: proteins were reduced and alkylated (15 mM TCEP, 30 mM CAA, 30min at RT in the dark) in the presence of urea 4 M and digested with trypsin in urea 1 M, 50 mM Tris pH 8 overnight at 37 °C

(Promega) at an estimated protein:enzyme ratio 1:100. FASP digestion: proteins were reduced and alkylated (15 mM TCEP, 30 mM CAA, 30 min in the dark, RT) and sequentially digested with chymotrypsin (SigmaAldrich) (protein:enzyme ratio 1:100, o/n at RT). In all cases, digestion was quenched by adding 0.1% TFA and resulting peptides were desalted using C18 stage-tips. LC-MS/MS was done by coupling an UltiMate 3000 RSLCnano LC system to a Q Exactive Plus mass spectrometer (Thermo Fisher Scientific). 2 to 5 ml of peptides were loaded into a trap column (Acclaim™ PepMap™ 100 C18 LC Columns 5 µm, 20 mm length) for 3 min at a flow rate of 10 µl/min in 0.1% formic acid. Then, peptides were transferred to an EASY-Spray PepMap RSLC C18 column (Thermo) (2 µm, 75 µm x 50 cm) operated at 45 °C and separated using a 60 min effective gradient (buffer A: 0.1% FA; buffer B: 100% ACN, 0.1% FA) at a flow rate of 250 nl/min. The gradient used was, from 4% to 6% B in 2 min, from 6% to 33% B in 58 minutes, plus 10 additional minutes at 98% B. Peptides were sprayed at 1.5 kV into the mass spectrometer via the EASY-Spray source. The capillary temperature was set to 300 °C. The mass spectrometer was operated in a data-dependent mode, with an automatic switch between MS and MS/MS scans using a top 15 method (intensity threshold ≥ 4.5 x 10^4, dynamic exclusion of 5 or 10 sec and excluding charges unassigned, +1 and > +6). MS spectra were acquired from 350 to 1500 m/z with a resolution of 70,000 FWHM (200 m/z). Ion peptides were isolated using a 2.0 Th window and fragmented using higher-energy collisional dissociation (HCD) with a normalized collision energy of 27. MS/MS spectra resolution was set to 35,000 (200 m/z). The ion target values were 3 x 10^6 for MS (maximum IT of 25 ms) and 10^5 for MS/MS (maximum IT of 110 msec). Raw files were processed with Maxquant (v 1.6 and higher) using the standard settings against the corresponding sequences of the expressed recombinant proteins, and an E. coli protein database (UniProtKB/Swiss-Prot, 20,373 sequences) supplemented with contaminants. Carbamidomethylation of cysteines was set as a fixed modification whereas oxidation of methionines, protein N-term acetylation and phosphorylation of serines, threonines and tyrosines were set as variable modifications.

Minimal peptide length was set to 7 amino acids and a maximum of two tryptic missed-cleavages were allowed. Results were filtered at 0.01 FDR (peptide and protein level). Raw data were imported into Skyline. Label free quantification of identified phosphopeptides was performed using the extracted ion chromatogram of the isotopic distribution. Only peaks without interference were used for quantification. Phosphopeptides intensities were normalized by the intensity of non-modified peptides from the target protein.

Differential Scanning Fluorometry (DSF)

To evaluate the thermal stability of recombinant c-Src WT and indicated variants in the absence of (apo) and in complex with Ponatinib, we applied two different scanning fluorometry methods. First, an indirect SYPRO Orange-based method. For this assay the total reaction volume was adjusted to 40 µl at 1-2 µM protein, 10 µM inhibitor, and 2 x SYPRO Orange concentrations subjected to a gradient of temperature from 20 to 95 °C. Fluorescence was measured on an Applied Biosystem 7300 Real-Time PCR system. Second, a direct method based on changes in intrinsic fluorescence upon a quick gradient of temperature was measured using a tycho instrument (Nanotemper) at 1-2 µM protein, 10 µM inhibitor concentration and following manufacturer´s instructions.

Drosophila strains

Drosophila melanogaster strains were maintained and raised at 25ºC under standard conditions. The stocks used, described in in Flybase (http://flybase.org/), are the following ones: GMRGal4 (eye expression, BSDC 9146), salEPvGal4 (wing expression, BDSC 80573), btlGal4 (tracheal expression, gift of S. Hayashi), src^{F80}/CyOlacZ; UASSrc/TM6 dfdYFP and UASSrc-KM (gift of S. Luschnig); UASSrcY400F/TM6 dfdYFP (this work). Balancer chromosomes CyO, TM3 or TM6 marked with LacZ, GFP or YFP were used to follow the mutations and constructs of interest in the different chromosomes. Transgenes were generated by injecting the pUASTattBSrc42aY400F

construct to obtain directed insertions at 68E by the “Transgenesis Service” of the “Centro de Biología Molecular Severo Ochoa” (CBM, Madrid). Transgene expression was achieved using the Gal4/UAS system [33] at 25°C (GMRGal4) or 29°C (btlGal4 and salEPvGal4).

Immunohistochemistry

Embryos were stained following standard protocols. Embryos were fixed in 4% formaldehyde (Sigma-Aldrich) in PBS1x-Heptane (1:1) for 20 min. Embryos transferred to new tubes were washed in PBT-BSA blocking solution and shaken in a rotator device at room temperature. Embryos were incubated with the primary antibodies in PBT-BSA overnight at 4 ºC. Secondary antibodies diluted in PBT-BSA (and for the CBP staining) were added after washing and were incubated at room temperature for 2-5 h in the dark. Embryos were washed, mounted on microscope glass slides with Fluoromount-G (Southern Biotech) and covered with thin glass slides. Primary antibodies used were goat anti-GFP (1:600) from Roche and chicken anti-β-gal (1:200) from Abcam. Alexa Fluor 488, 555, 647 (Invitrogen) secondary antibodies were used at 1:300 in PBT 0.5% BSA. CBP (Chitin Binding Protein, produced by N. Martín in Dr. Casanova's lab, New England Biolabs Protocol) was used as a secondary antibody at 1:300 to detect chitin and visualize the tracheal branches.

Image acquisition

Images from fixed embryos were taken using Leica TCS-SPE with the 20x and 63x immersion oil (1.40-0.60; Immersol 518F – Zeiss oil) objectives and additional zoom. Settings were adjusted for the different channels prior to image acquisition. Z-stack sections of 0.24-0.5 μm were acquired. The images were imported and processed using Fiji (ImageJ 1.49b) and Photoshop for measurements and adjustments, and assembled into figures using Illustrator. Images from adults were obtained with an Olympus MVX10 macroscope using EFI (extended focus imaging) at the ADM facility of IRB-PCB.

Morphometric analyses

Confocal projections were used to analyze the length of the embryonic dorsal trunk (DT) stained with CBP in stage 16 embryos. We traced the path using the freehand line selection tool of Fiji (ImageJ) software between the junction DT/Transverse Connective (TC) from metamere 2 to 9 following the DT curvature. DT length was expressed as the ratio between the DT path and the length of the embryo. Data from quantifications was imported and treated in the Excel software and in GraphPad Prism 9.0.0, where graphics were finally generated. Graphics shown are scatter dot plots, where bars indicate the mean and the standard deviation. Statistical analyses comparing the mean of two groups of quantitative continuous data were performed in GraphPad Prism 9.0.0 using unpaired two-tailed student's t-test applying Welch's correction. Differences were considered significant when $p < 0.05$. *$p < 0.05$, **$p < 0.01$, *** $p < 0.001$, **** $p < 0.0001$ where n.s. means not statistically significant.

Crystallization, Diffraction, Data Collection, and Processing

Crystals of the c-Src KD (aa 252-536, human) in complex with ponatinib were obtained by mixing recombinant protein and ponatinib in a 1:2 molar ratio. Sample was concentrated using an Amicon Ultra 10K molecular weight cut-off centrifugal filter device (Merck Millipore, Billerica, Massachusetts, USA) up to 8 mg/ml. The final protein concentration was determined by UV spectroscopy (Nanodrop one, Thermo Scientific).

Crystallization for c-Src KD in complex with ponatinib were performed by sitting-drop vapour diffusion at 20 ºC in MRC-2 crystallization plates (Molecular Dimensions, Newmarket, Suffolk, England) by mixing 0.5 μl of protein-ligand solution at 8 mg/ml with 0.5 μL of reservoir solution. After several days some crystals were obtained in a drop containing 50 mM sodium acetate pH 4.6, 100 mM sodium chloride, 20% w/v PEG 4000 and 10% v/v 2-Propanol. A cryoprotectant solution consisting of the reservoir solution including 20% (v/v) glycerol was used to freeze the crystals and mounted in LithoLoops (Molecular Dimensions, Newmarket, England) prior to vitrification in liquid nitrogen for

data collection. X-ray diffraction data were collected at a wavelength of 0.98 Å on beamline XALOC-BL13 of the ALBA Synchrotron Light Facility (Barcelona, Spain) using a Pilatus 6M pixel detector (Dectris Ltd, Baden, Switzerland). Crystals were kept at 100 K during data collection. Reflections were integrated with the programme iMOSFLM and reduced using POINTLESS, AIMLESS and TRUNCATE, all integrated in the Collaborative Computational Project Number 4 (CCP4). Molecular replacement was carried out using the atomic coordinates of c-Src KD in complex with imatinib (PDB:3EL8) using PHASER [34]. Adjustment of the model was performed with COOT [35] and refinement was carried out with Refmac5 [36] applying twin refinement amplitude-based settings. Model validation was carried out with MOLPROBITY and structure figures were made using the graphics program PYMOL (http://www.pymol.org) (Schrödinger LLC, Cambridge MA, U.S.A.). The crystallographic coordinates and structure factors for the crystal structure of human c-Src KD in complex with Ponatinib reported in this paper is PDB: 7OTE. For data statistics, see table 1.

Small angle X-ray scattering (SAXS)

SAXS experiments were conducted at the beamline B21 of the Diamond Light Source (Didcot, UK) [37]. A sample of 40 ul of Src WT (3D-construct) at concentration of 3 mg/ml was delivered at 20 ºC via an in-line Agilent 1200 HPLC system in a Superdex 200 Increase 3.2/300 column (Cytiva), using a running buffer composed by 20 mM Tris pH 8.0, 150 mM NaCl, 1% glycerol and 1 mM DTT. The continuously eluting samples were exposed for 20 s and a total number of 599 frames recorded, using an X-ray wavelength of 1 Å, and a sample to detector (Eiger 4M) distance of 3.6 m. The frames recorded immediately before elution of the sample were subtracted from the protein scattering profiles. The Scåtter software package (www.bioisis.net) was used to analyse data, buffer-subtraction, scaling, merging and checking possible radiation damage of the samples. The R_g value was calculated with the Guinier approximation assuming that at very small angles $q < 1.3/R_g$. The particle distance distribution, D_{max}, was calculated from

the scattering pattern with GNOM, and shape estimation was carried out with DAMMIF/DAMMIN, all these programs included in the ATSAS package [38]. The protein molecular mass was estimated with GNOM. A generated PDB-based homology models were made using the program COOT by manually adjusting the X-ray structures obtained in this work, into the envelope given by SAXS until a good correlation between the real-space scattering profile calculated for the homology model matched the experimental scattering data. This was computed with the program FoXS [39]

Molecular dynamics simulations

The crystal structure of the active Src dimer presented in this study (PDB ID: 7OTE) was used as a starting conformation for molecular dynamics simulations. The disordered residues in the activation loop (410-426) were modeled using a crystal structure of Src in the active conformation (PDB ID: 1YI6). The dimer model was minimized using RosettaRelax [40] with all atom constraints to the native coordinates. On the activation loop, Tyr416 was modeled as phospho-tyrosine using PyTMs [41]. In the kinase active site, we replaced the Ponatinib with an ATP and 2 magnesium ions. These modifications were all applied to both monomers. Unbiased all-atom molecular dynamics simulations were performed using GROMACS 2021.4 [42]. Structures were parameterized using the CHARMM36 [43] force field and solvated with the TIP3P water model. Random solvents molecules were replaced with sodium or chloride ions to neutralize the charge of the system and bring the concentration to 0.1 mol/L. The system was contained in a dodecahedron at least 1 nm larger than the protein from all sides with periodic boundary conditions. Long-range interactions were calculated with particle mesh Ewald. Neighbor lists were maintained using the Verlet cutoff scheme. The system underwent steepest descent minimization until the maximum force was <100 kJ/mol. Canonical ensemble [44] was used to heat the system from 0 to 310 K in 100 ps. Isothermal–isobaric ensemble [45](1 bar, 310 K) was applied for 100 ps. Positional restraints were applied during equilibration. Production runs used 2 fs time steps.

4. SUPPLEMENTRY REFERENCES

1. Johnson H, Lescarbeau RS, Gutierrez JA, White FM. Phosphotyrosine profiling of NSCLC cells in response to EGF and HGF reveals network specific mediators of invasion. J Proteome Res 12, 1856-1867 (2013).

2. Broome MA, Hunter T. Requirement for c-Src catalytic activity and the SH3 domain in platelet-derived growth factor BB and epidermal growth factor mitogenic signaling. J Biol Chem 271, 16798-16806 (1996).

3. Broome MA, Hunter T. The PDGF receptor phosphorylates Tyr 138 in the c-Src SH3 domain in vivo reducing peptide ligand binding. Oncogene 14, 17-34 (1997).

4. Gu TL, et al. Survey of tyrosine kinase signaling reveals ROS kinase fusions in human cholangiocarcinoma. PLoS One 6, e15640 (2011).

5. Guo A, et al. Signaling networks assembled by oncogenic EGFR and c-Met. Proc Natl Acad Sci U S A 105, 692-697 (2008).

6. Ushio-Fukai M, Griendling KK, Becker PL, Hilenski L, Halleran S, Alexander RW. Epidermal growth factor receptor transactivation by angiotensin II requires reactive oxygen species in vascular smooth muscle cells. Arterioscler Thromb Vasc Biol 21, 489-495 (2001).

7. Vadlamudi RK, Sahin AA, Adam L, Wang RA, Kumar R. Heregulin and HER2 signaling selectively activates c-Src phosphorylation at tyrosine 215. FEBS Lett 543, 76-80 (2003).

8. Xie Y, Zhang D, Jarori GK, Davisson VJ, Ben-Amotz D. The Raman detection of peptide tyrosine phosphorylation. Anal Biochem 332, 116-121 (2004).

9. Yang Z, et al. The epidermal growth factor receptor tyrosine kinase inhibitor ZD1839 (Iressa) suppresses c-Src and Pak1 pathways and invasiveness of human cancer cells. Clin Cancer Res 10, 658-667 (2004).

10. Barker SC, Kassel DB, Weigl D, Huang X, Luther MA, Knight WB. Characterization of pp60c-src tyrosine kinase activities using a continuous assay: autoactivation of the enzyme is an intermolecular autophosphorylation process. Biochemistry 34, 14843-14851 (1995).

11. Chan GK, McGrath JA, Parsons M. Spatial activation of ezrin by epidermal growth factor receptor and focal adhesion kinase co-ordinates epithelial cell migration. Open Biol 11, 210166 (2021).

12. Dong J, et al. MG53 inhibits angiogenesis through regulating focal adhesion kinase signalling. J Cell Mol Med 25, 7462-7471 (2021).

13. Jayabal P, et al. NELL2-cdc42 signaling regulates BAF complexes and Ewing sarcoma cell growth. Cell Rep 36, 109254 (2021).

14. Liu Z, et al. Melatonin potentiates the cytotoxic effect of Neratinib in HER2(+) breast cancer through promoting endocytosis and lysosomal degradation of HER2. Oncogene 40, 6273-6283 (2021).

15. Tognoli ML, et al. RASSF1C oncogene elicits amoeboid invasion, cancer stemness, and extracellular vesicle release via a SRC/Rho axis. EMBO J 40, e107680 (2021).

16. Wang S, et al. CCM3 is a gatekeeper in focal adhesions regulating mechanotransduction and YAP/TAZ signalling. Nat Cell Biol 23, 758-770 (2021).

17. Wu Y, et al. HNRNPH1-stabilized LINC00662 promotes ovarian cancer progression by activating the GRP78/p38 pathway. Oncogene 40, 4770-4782 (2021).

18. Bai Y, et al. Phosphoproteomics identifies driver tyrosine kinases in sarcoma cell lines and tumors. Cancer Res 72, 2501-2511 (2012).

19. DeNardo BD, et al. Quantitative phosphoproteomic analysis identifies activation of the RET and IGF-1R/IR signaling pathways in neuroblastoma. PLoS One 8, e82513 (2013).

20. Helou YA, Nguyen V, Beik SP, Salomon AR. ERK positive feedback regulates a widespread network of tyrosine phosphorylation sites across canonical T cell signaling and actin cytoskeletal proteins in Jurkat T cells. PLoS One 8, e69641 (2013).

21. Palacios-Moreno J, et al. Neuroblastoma tyrosine kinase signaling networks involve FYN and LYN in endosomes and lipid rafts. PLoS Comput Biol 11, e1004130 (2015).

22. Sharma K, et al. Ultradeep human phosphoproteome reveals a distinct regulatory nature of Tyr and Ser/Thr-based signaling. Cell Rep 8, 1583-1594 (2014).

23. Tzouros M, et al. Development of a 5-plex SILAC method tuned for the quantitation of tyrosine phosphorylation dynamics. Mol Cell Proteomics 12, 3339-3349 (2013).

24. Yoshida T, et al. Tyrosine phosphoproteomics identifies both codrivers and cotargeting strategies for T790M-related EGFR-TKI resistance in non-small cell lung cancer. Clin Cancer Res 20, 4059-4074 (2014).

25. Zhou H, et al. Toward a comprehensive characterization of a human cancer cell phosphoproteome. J Proteome Res 12, 260-271 (2013).

26. Advani G, et al. Csk-homologous kinase (Chk) is an efficient inhibitor of Src-family kinases but a poor catalyst of phosphorylation of their C-terminal regulatory tyrosine. Cell Commun Signal 15, 29 (2017).

27. Chu CT, Chen YH, Chiu WT, Chen HC. Tyrosine phosphorylation of lamin A by Src promotes disassembly of nuclear lamina in interphase. Life Sci Alliance 4, (2021).

28. Ci S, et al. Src-mediated phosphorylation of GAPDH regulates its nuclear localization and cellular response to DNA damage. FASEB J 34, 10443-10461 (2020).

29. Fang XQ, et al. Focal adhesion kinase regulates the phosphorylation protein tyrosine phosphatase-alpha at Tyr789 in breast cancer cells. Mol Med Rep 11, 4303-4308 (2015).

30. Gujral TS, Chan M, Peshkin L, Sorger PK, Kirschner MW, MacBeath G. A noncanonical Frizzled2 pathway regulates epithelial-mesenchymal transition and metastasis. Cell 159, 844-856 (2014).

31. Trewhella J, Jeffries CM, Whitten AE. 2023 update of template tables for reporting biomolecular structural modelling of small-angle scattering data. Acta Crystallogr D Struct Biol 79, 122-132 (2023).

32. Seeliger MA, et al. High yield bacterial expression of active c-Abl and c-Src tyrosine kinases. Protein Sci 14, 3135-3139 (2005).

33. Brand AH, Perrimon N. Targeted gene expression as a means of altering cell fates and generating dominant phenotypes. Development 118, 401-415 (1993).

34. McCoy AJ. Solving structures of protein complexes by molecular replacement with Phaser. Acta Crystallogr D Biol Crystallogr 63, 32-41 (2007).

35. Emsley P, Lohkamp B, Scott WG, Cowtan K. Features and development of Coot. Acta Crystallogr D Biol Crystallogr 66, 486-501 (2010).

36. Murshudov GN, et al. REFMAC5 for the refinement of macromolecular crystal structures. Acta Crystallogr D Biol Crystallogr 67, 355-367 (2011).

37. Cowieson NP, et al. Beamline B21: high-throughput small-angle X-ray scattering at Diamond Light Source. J Synchrotron Radiat 27, 1438-1446 (2020).

38. Petoukhov MV, et al. New developments in the ATSAS program package for small-angle scattering data analysis. J Appl Crystallogr 45, 342-350 (2012).

39. Schneidman-Duhovny D, Hammel M, Tainer JA, Sali A. FoXS, FoXSDock and MultiFoXS: Single-state and multi-state structural modeling of proteins and their complexes based on SAXS profiles. Nucleic Acids Res 44, W424-429 (2016).

40. Conway P, Tyka MD, DiMaio F, Konerding DE, Baker D. Relaxation of backbone bond geometry improves protein energy landscape modeling. Protein Sci 23, 47-55 (2014).

41. Warnecke A, Sandalova T, Achour A, Harris RA. PyTMs: a useful PyMOL plugin for modeling common post-translational modifications. BMC Bioinformatics 15, 370 (2014).

42. Abraham MJ, Gready JE. Optimization of parameters for molecular dynamics simulation using smooth particle-mesh Ewald in GROMACS 4.5. J Comput Chem 32, 2031-2040 (2011).

43. Huang J, MacKerell AD, Jr. CHARMM36 all-atom additive protein force field: validation based on comparison to NMR data. J Comput Chem 34, 2135-2145 (2013).

44. Bussi G, Donadio D, Parrinello M. Canonical sampling through velocity rescaling. J Chem Phys 126, 014101 (2007).

45. Eslami H, Mozaffari F, Moghadasi J, Muller-Plathe F. Molecular dynamics simulation of confined fluids in isosurface-isothermal-isobaric ensemble. J Chem Phys 129, 194702 (2008).
